# Novel Anaplasmataceae agents *Candidatus* Ehrlichia hydrochoerus and *Anaplasma* spp. Infecting Capybaras, Brazil

**DOI:** 10.3201/eid2802.210705

**Published:** 2022-02

**Authors:** Thállitha S.W.J. Vieira, Flávia C.M. Collere, Larissa D.R. Ferrari, Rafael A. Baggio, Rogério R. Lange, Marcos V. Ferrari, Juan C.M. Duque, Gustavo S. Sanches, Nathália A. Pereira, Daniel M. Aguiar, Marcelo B. Labruna, Rafael F.C. Vieira

**Affiliations:** Universidade Federal do Paraná, Curitiba, Brazil (T.S.W.J. Vieira, F.C.M. Collere, L.D.R. Ferrari, R.R. Lange, M.V. Ferrari, J.C.M. Duque, R.F.C. Vieira);; Universidade Federal de Minas Gerais, Belo Horizonte, Brazil (R.A. Baggio);; Pontifícia Universidade Católica do Paraná, Curitiba (G.S. Sanches);; Universidade Federal do Mato Grosso, Cuiabá, Brazil (N.A. Pereira, D.M. Aguiar);; Universidade de São Paulo, São Paulo, Brazil (M.B. Labruna);; Global One Health initiative, The Ohio State University, Columbus, Ohio, USA (R.F.C. Vieira)

**Keywords:** Anaplasma, novel Anaplasmataceae agents, Hydrochoerus hydrochaeris, tickborne diseases, vector-borne infections, Candidatus Ehrlichia hydrochoerus, Anaplasma spp., capybaras, Brazil, bacteria

## Abstract

We amplified *Ehrlichia* and *Anaplasma* DNA from *Amblyomma dubitatum* tick–infested capybaras (*Hydrochoerus hydrochaeris*) in southern Brazil. Sequencing of 16S rRNA, *sodB*, and *groEL* indicated a novel *Ehrlichia* species, and sequencing of 16S rRNA from 2 capybaras indicated a novel *Anaplasma* species. The tick vectors remain unknown.

*Ehrlichia* and *Anaplasma* species are tickborne bacteria that infect animals and humans worldwide. To date, 6 *Ehrlichia* species have been described (*E. canis*, *E. chaffeensis*, *E. ewingii*, *E. muris*, *E. ruminantium*, and *E. minasensis*), and 8 *Anaplasma* species have been described (*A. bovis*, *A. capra*, *A. centrale*, *A. marginale*, *A. odocoilei*, *A. ovis*, *A. platys*, and *A. phagocytophilum*). In addition, other native *Ehrlichia* species have been described in wild animals from Brazil (*1*).

Although capybaras (*Hydrochoerus hydrochaeris*), the largest living rodents in the world, have been implicated as a major amplifying host of *Rickettsia rickettsii* (the etiologic agent of Brazilian spotted fever) for *Amblyomma sculptum* ticks, studies focusing on other tickborne diseases agents are lacking in this rodent. Accordingly, we conducted a comprehensive survey for the detection of *Ehrlichia* and *Anaplasma* species in a population of capybaras from Pinhais Municipality, Paraná State, southern Brazil.

We retrieved blood samples from 17 capybaras and salivary glands from 11 *Amblyomma dubitatum* ticks from these capybaras that were collected for a previous study conducted in southern Brazil (*2*). We screened blood samples by using PCR targeting of the 16S rRNA gene of *Ehrlichia* and *Anaplasma* (*3*,*4*). We then tested samples positive by PCR by using PCR that targeted a fragment of the *dsb* and *sodB* genes of *Ehrlichia* species (*1*,*5*) and the *groEL* gene of *Ehrlichia* and *Anaplasma* species (*6*). We used blood samples from dogs positive for *E. canis* as positive controls and nuclease-free water samples as negative controls.

The *Ehrlichia* 16S rRNA PCR assay yielded amplicons in 16/17 (94.12% [95% CI 73.02%–98.95%]) capybaras, from which we generated amplicons by the *sodB* PCR (300 bp) and *groEL* PCR (1,100 bp) assays. No sample yielded amplicon by the *dsb* PCR assay. We sequenced amplicons obtained from 4 16S rRNA, 5 *sodB*, and 4 *groEL* PCR-positive samples in both directions by using the Sanger method. We submitted all nucleotide sequences obtained to GenBank (Appendix).

We observed infestations by *A. dubitatum* ticks in all capybaras, from which we collected 26 males, 16 females, and 122 nymphs. Among salivary glands from 11 adult ticks, 1 (9.09%) tested positive for *Ehrlichia* species by the 16S rRNA PCR. However, multiple attempts to sequence the 16S rRNA gene detected in tick salivary glands were unsuccessful because of the faint bands.

We observed neither abnormalities nor inclusion-like bodies of *Ehrlichia* or *Anaplasma* during the evaluation of Giemsa-stained thin blood smears of the capybaras. We tested *Ehrlichia* antibodies in capybara serum samples with an indirect immunofluorescent assay using *E. canis* (São Paulo and Cuiabá strains) as antigens; serum samples were positive if reacting at a dilution >1:40 (*7*). A total of 6/17 (35.29% [95% CI 17.31%–58.70%]) capybaras showed antibodies against >1 of the *E. canis* antigens. When we used the Cuiabá strain of *E. canis* as antigen, 4/17 (23.53% [95% CI 9.56%–47.26%]) capybaras were seropositive, whereas 6/17 (35.29%) were positive when we used the São Paulo strain. Four capybaras were seropositive for both *E. canis* strains. Antibody endpoint titers ranged from 40 to 640 for both *E. canis* antigens.

According to serologic testing, PCR amplification, and DNA sequencing results, *A. dubitatum* tick–infested capybaras in southern Brazil may be infected with a novel *Ehrlichia* agent and a novel *Anaplasma* species. Serologic screening showed exposure to *Ehrlichia* species in 35% of the capybaras. A previous study failed to detect *Ehrlichia* DNA in spleen tissue of capybaras from southeastern Brazil (*8*), and we know of no previous study of *Anaplasma* species that has been performed in this rodent species.

Partial sequences of 16S rRNA and 2 protein-coding genes (*sodB* and *groEL*) obtained from capybaras indicate a novel *Ehrlichia* species. Partial 16S rRNA gene sequences from capybara no. II showed that the detected *Ehrlichia* agent shared 95.67% identity with *A. phagocytophilum*, whereas sequences from capybara no. III showed that the detected *Ehrlichia* agent shared 94.28% identity with *E. chaffeensis*. Partial *sodB* genes showed 82.23%–85.07% identity with *E. chaffeensis* or *E. ruminantium*, whereas partial *groEL* genes showed identity with 76.52% with *A. phagocytophilum*. A previous study stated that different bacterial isolates showing <97% similarity in the 16S rRNA gene belong to different species (*9*). In addition, protein-coding genes should be used in addition to the 16S rRNA gene for identification of novel species (*10*). Our genetic findings support the infection of capybaras in Brazil with a novel *Ehrlichia* species, herein named *Candidatus* Ehrlichia hydrochoerus (Figure).

**Figure:**
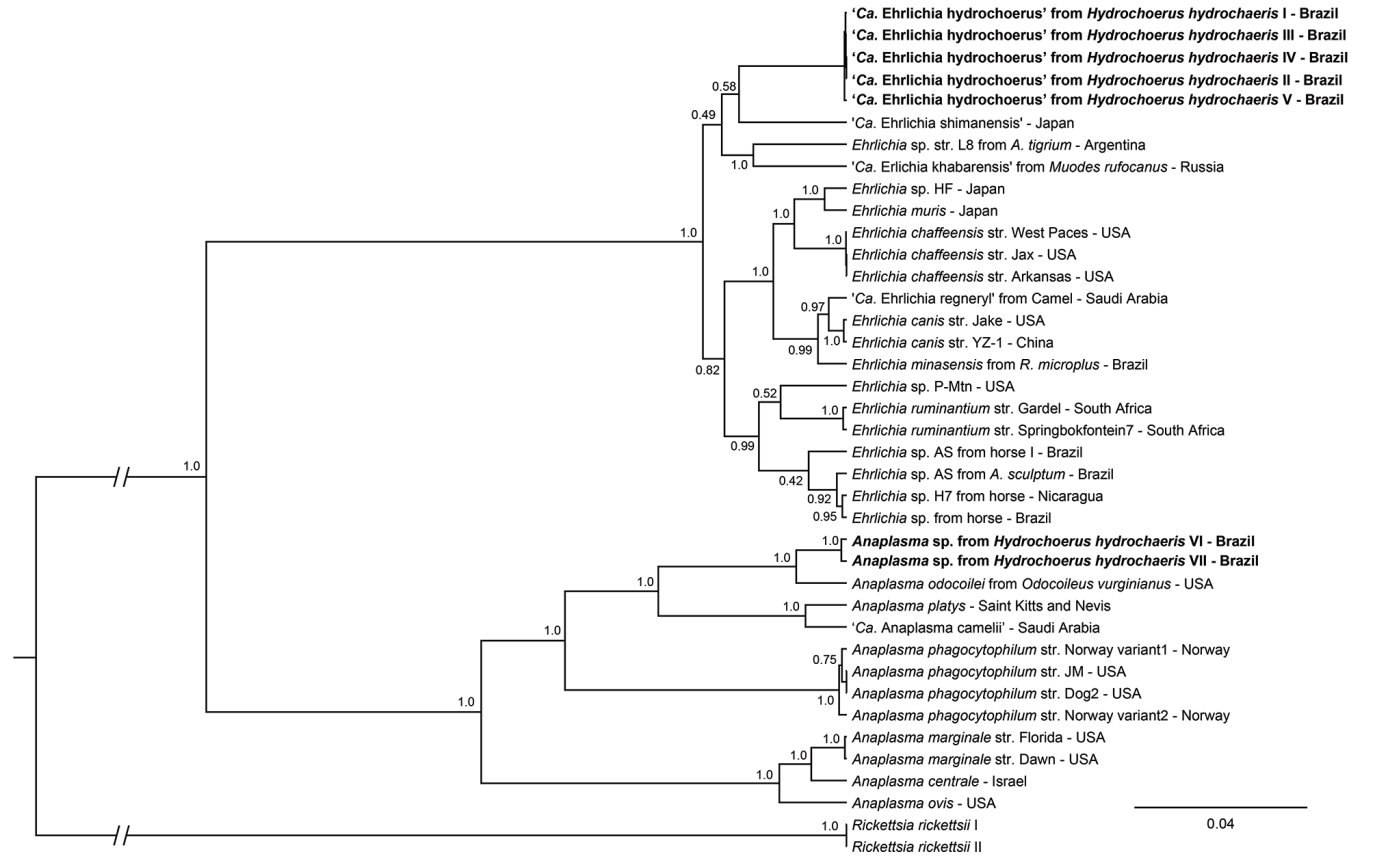
Phylogenetic analysis of 16S rRNA, *sodB*, and *groEL* partial sequences of *Candidatus* Ehrlichia hydrochoerus and *Anaplasma* spp. obtained from capybaras (*Hydrochoerus hydrochaeris*), southern Brazil. These sequences (in bold) and those of other *Ehrlichia* and *Anaplasma* species were aligned using MAFFT 7.110 (https://mafft.cbrc.jp/alignment/server). Phylogenetic analyses of each gene were based on Bayesian inference using Beast version 1.8.4 (https://beast.community/index.html). We performed 3 independent runs of 100 million generations of Monte Carlo Markov chain with 1 sampling/10,000 generations and a 10% burn-in. We estimated substitution models as generalized time reversible plus gamma for *16S rRNA* (A), Hasegawa–Kishino–Yano plus gamma for *sodB* (B), and Tamura–Nei plus gamma for *groEL* (C) genes on the basis of Akaike information criterion by using jModeltest version 2.1.10 (https://github.com/ddarriba/jmodeltest2/releases/tag/v2.1.10r20160303). The tree was rooted with *Rickettsia rickettsii* (GenBank accession nos. CP000766.3 and CP018913.1). Complete GenBank accession numbers are listed in the Appendix . Scale bar indicates number of substitutions per site. *Ca*., *Candidatus.*

Partial sequences of 16S rRNA gene obtained from capybaras VI and VII demonstrated a novel *Anaplasma* species. Partial 16S rRNA gene sequences showed identity of 96.76% with *Anaplasma* sp. detected in dogs from the Philippines and 97.93% with *A. phagocytophilum*, with 100% query coverage. Bayesian inference showed that the capybara *Anaplasma* species detected was related to *A. odocoilei* from North America, which indicates a novel *Anaplasma* species infecting capybaras in Brazil.

Appendix Additional information about novel Anaplasmataceae agents *Candidatus* Ehrlichia hydrochoerus and *Anaplasma* spp., infecting capybaras, Brazil.
