# Supplementary material for: Novel Anaplasmataceae agents Candidatus Ehrlichia hydrochoerus and Anaplasma spp. Infecting Capybaras, Brazil
Source: Emerg Infect Dis. 2022 Feb;28(2):480–2. doi: 10.3201/eid2802.210705 (PMC8798694; doi:10.3201/eid2802.210705)
Supplement: Appendix — Additional information about novel Anaplasmataceae agents Candidatus Ehrlichia hydrochoerus and Anaplasma spp., infecting capybaras, Brazil. [file 21-0705-Techapp-s1.pdf]

Article DOI: https://doi.org/10.3201/eid2802.210705

# Novel Anaplasmataceae Agents *Candidatus* Ehrlichia hydrochoerus and *Anaplasma* spp. Infecting Capybaras Brazil

## Appendix

**Appendix Table.** *Anaplasma* and *Ehrlichia* species and their GenBank accession numbers used for the Bayesian phylogenetic tree*

| Species | 16S | GroEL | sodB |
|---|---|---|---|
| *Anaplasma* sp. from *H. hydrochaeris* VI - Brazil | MW785881.1 | *NA* | *NA* |
| *Anaplasma* sp. from *H. hydrochaeris* VII - Brazil | MW785882.1 | *NA* | *NA* |
| '*Ca*. Ehrlichia hydrochoerus' from *H. hydrochaeris* I - Brazil | *NA* | MW816651.1 | OK236545.1 |
| '*Ca*. Ehrlichia hydrochoerus' from *H. hydrochaeris* II - Brazil | MW785879.1 | *NA* | MW816538.1 |
| '*Ca*. Ehrlichia hydrochoerus' from *H. hydrochaeris* III - Brazil | MW785880.1 | MW816652.1 | MW816537.1 |
| '*Ca*. Ehrlichia hydrochoerus' from *H. hydrochaeris* IV - Brazil | *NA* | MW816653.1 | MW816536.1 |
| '*Ca*. Ehrlichia hydrochoerus' from *H. hydrochaeris* V - Brazil | *NA* | MW816654.1 | MW816535.1 |
| '*Ca*. Anaplasma camelii' - Saudi Arabia | KF843825.1 | *NA* | *NA* |
| *Anaplasma centrale* - Israel | CP001759.1 | CP001759.1 | CP001759.1 |
| *Anaplasma marginale* str. Dawn - USA | CP006847.1 | CP006847.1 | CP006847.1 |
| *Anaplasma marginale* str. Florida - USA | CP001079.1 | CP001079.1 | CP001079.1 |
| *Anaplasma odocoilei* from *O. vurginianus* - USA | JX876644.1 | JX876642.1 | *NA* |
| *Anaplasma ovis* - USA | CP015994.2 | CP015994.2 | CP015994.2 |
| *Anaplasma phagocytophilum* str. Dog2 - USA | CP006618.1 | CP006618.1 | CP006618.1 |
| *Anaplasma phagocytophilum* str. JM - USA | CP006617.1 | CP006617.1 | CP006617.1 |
| *Anaplasma phagocytophilum* str. Norway variant1 - Norway | CP046639.1 | CP046639.1 | CP046639.1 |
| *Anaplasma phagocytophilum* str. Norway variant2 - Norway | CP015376.1 | CP015376.1 | CP015376.1 |
| *Anaplasma platys* - Saint Kitts and Nevis | CP046391.1 | CP046391.1 | CP046391.1 |
| '*Ca*. Erlichia khabarensis' from *M. rufocanus* - Russia | *NA* | FJ966351.1 | *NA* |
| '*Ca*. Ehrlichia regneryl' from Camel - Saudi Arabia | KF843826.1 | *NA* | *NA* |
| '*Ca*. Ehrlichia shimanensis' - Japan | *NA* | AB074462.1 | *NA* |
| *Ehrlichia canis* str. Jake - USA | CP000107.1 | CP000107.1 | CP000107.1 |
| *Ehrlichia canis* str. YZ-1 - China | CP025749.1 | CP025749.1 | CP025749.1 |
| *Ehrlichia chaffeensis* str. Arkansas - USA | CP000236.1 | CP000236.1 | CP000236.1 |
| *Ehrlichia chaffeensis* str. Jax - USA | CP007475.1 | CP007475.1 | CP007475.1 |
| *Ehrlichia chaffeensis* str. West Paces - USA | CP007480.1 | CP007480.1 | CP007480.1 |
| *Ehrlichia minasensis* from *R. microplus* - Brazil | CDGH01000025.1 | QOHL01000018.1 | CDGH01000066.1 |
| *Ehrlichia muris* - Japan | CP006917.1 | CP006917.1 | CP006917.1 |
| *Ehrlichia ruminantium* str. Gardel - South Africa | CR925677.1 | CR925677.1 | CR925677.1 |
| *Ehrlichia ruminantium* str. Springbokfontein7 - South Africa | CP040111.1 | CP040111.1 | CP040111.1 |
| *Ehrlichia* sp. AS from *A. sculptum* - Brazil | *NA* | *NA* | MW070031.1 |
| *Ehrlichia* sp. AS from horse I - Brazil | MT514732.1 | MT191353.1 | *NA* |
| *Ehrlichia* sp. from horse - Brazil | *NA* | MG385128.1 | MG385129.1 |
| *Ehrlichia* sp. H7 from horse - Nicaragua | KJ434178.1 | KJ434179.1 | KJ434180.1 |
| *Ehrlichia* sp. HF - Japan | CP007474.1 | CP007474.1 | CP007474.1 |
| *Ehrlichia* sp. P-Mtn - USA | *NA* | *NA* | KC702804.1 |
| *Ehrlichia* sp. str. L8 from *A. tigrium* - Argentina | *NA* | MN266482.1 | *NA* |

*NA, not available (missing sequences).
